# Prevalence of work-related musculoskeletal symptoms and associated risk factors among domestic gas workers and staff of works department in Enugu, Nigeria: a cross-sectional study

**DOI:** 10.1186/s12891-020-03615-5

**Published:** 2020-09-01

**Authors:** Chinenye Doris Oluka, Esther Obidike, Antoninus Obinna Ezeukwu, Ogochukwu Kelechi Onyeso, Echezona Nelson Dominic Ekechukwu

**Affiliations:** 1 Physiotherapy Department, Medical Center, University of Nigeria Enugu Campus, Enugu, Nigeria; 2 Department of Medical Rehabilitation, Faculty of Health Sciences and Technology, College of Medicine, University of Nigeria, Enugu, Nigeria; 3 Department of Physiotherapy, Nnamdi Azikiwe University Teaching Hospital, Nnewi, Nigeria; 4 Department of Physiotherapy, Faculty of Health Sciences, Bayelsa Medical University, Yenagoa, Nigeria; 5 LANCET Physiotherapy Wellness and Research Centre, Enugu, Nigeria

**Keywords:** Ergonomics, Exercise, Manual handling, Personal protective equipment, Prevalence, Shift duty, Musculoskeletal disorders

## Abstract

**Background:**

The impact of work-related musculoskeletal symptoms (WMSS) permeates various occupations.

**Objective:**

To compare WMSS and associated risk factors among domestic gas workers (DGWs) and staff of Works Department (SWD) in Enugu.

**Methods:**

One-hundred adults (DGW = 50, SWD = 50) participated in this cross-sectional study. The Nordic Musculoskeletal Questionnaire and a demographics questionnaire were used to assess the prevalence of WMSS and related risk factors. Data were analysed using independent *t*-test or Mann-Whitney U, chi-square, and logistic regression at *p* < 0.05.

**Results:**

The DGWs (86%) had a significantly (χ2 = 24.45, *p* < 0.001) higher WMSS than the SWD (38%). Lower-back (54%) and shoulder (52%) were the most affected body parts among the DGWs in comparison to the hips/thighs (20%) among the SWD. Work-related factors such as daily work-duration (χ2 = 75.44, *p* < 0.001), lifting training (χ2 = 96.24, *p <* 0.001), and use of personal protective equipment (PPE) of facemask (χ2 = 100.0, *p <* 0.001) and gloves (χ2 = 96.09, *p <* 0.001) were significantly associated with general WMSS among the DGWs. However, diastolic blood pressure (DBP) (OR = 1.29, *p* = 0.018), work duration > 8 h/day (OR = 0.001, *p* = 0.028), female gender (OR = 6.98–10.26, *p* < 0.05), sleep duration < 6 h/day (OR = 0.56–0.73, *p <* 0.05) and poor exercise behaviour (OR = 0.15, *p* = 0.013) were the identified independent risk factors of WMSS among DGWs, while DBP (OR = 0.99, *p* = 0.012) and female gender (OR = 6.47, *p* = 0.032) were the only identified independent risk factors for SWD.

**Conclusion:**

WMSS is significantly higher among DGWs than the SWD. High DBP, female gender, working beyond 8 h per day, sleeping less than 6 h per day, and insufficient exercise increase the risks of WMSDs, especially among the DGWs. To mitigate the adverse effects of WMSDs, SWD and DGWs require break and leave periods, PPE and assistive devices, exercise, medical check-up, and workplace ergonomics.

## Background

Greater than 9% of the global adult population suffers from physical disabilities caused by musculoskeletal disorders (MSDs). This prevalence is higher among women and increases markedly with age, accounting for 2% of global economic disease burden [1]. MSDs are usually related to work and interfere with health status, quality of life, and work-efficiency among individuals [2]. Work-related musculoskeletal symptoms (WMSS) are among the most significant occupational hazards in many industries [3]. In addition, WMSS have negative socioeconomic impact on individuals, organisations, and the society [4]. The risk factors of WMSS can broadly be categorised into mechanical and psychosocial factors. The mechanically-induced WMSS is associated with poor working conditions among manual workers [5–7]. Work-related musculoskeletal symptoms can also be caused by cumulative task-related musculoskeletal trauma arising from manual handling activities [4, 8].

Domestic Gas Workers (DGW) in Enugu metropolis are mostly employed in privately-owned liquefied natural gas (methane) stations. Due to the continuous demand for liquefied natural gas because of its usefulness in domestic cooking, bakeries, restaurants, and medium-scale industries, DGWs have to work daily, usually without any shifts. Gas cylinder refilling is a strenuous job, mostly associated with forceful exertion [9, 10]. In addition, DGWs often engage in manual handling activities such as lifting, bending, carrying, and repetitive movements [6]. It involves unloading and conveyance of the empty gas cylinders to the point where it would be refilled, filling and weighing the gas cylinders, testing for any valve leakages, and retuning the filled cylinders back to the customers.

Similarly, Staff of Works Department (SWD) in many tertiary institutions in Enugu engage in manual material handling such as maintenance of automobile, mechanical, and electrical installations, sweeping, mowing of lawns, cleaning of offices, and hostels. The staff of the Works Department of the University of Nigeria Enugu Campus (UNEC) and University of Nigeria Teaching Hospital (UNTH) Enugu mainly perform manual handling jobs. Their job demands a high level of physical strength, postural adaptations, and a relatively lesser amount of rest, which makes them vulnerable to MSD [11]. In this study, the researchers hypothesised that the SWD in tertiary institutions are exposed to similar WMSS risk-factors as in the case of DGWs. Therefore, this study compared the prevalence of WMSS among DGWs in Enugu metropolis and age- and sex-matched SWD in two tertiary institutions (UNEC and UNTH) in Enugu, Nigeria.

## Methods

### Participants

This cross-sectional exploratory research involved 100 participants (16 females and 84 males) recruited from domestic gas stations in Enugu Metropolis and from the Works Department of UNEC and UNTH in Enugu between July 1 and August 23, 2019. Fifty DGWs were conveniently recruited using the eligibility criteria and classified according to their sex and age ranges with a class-width of 3 years. Correspondingly, 50 sex- and age-matched SWD were purposively recruited to match the sample of DGW. The matching protocol was adapted from a recently published study among diabetic patients [12]. All the participants met the inclusion criteria and provided written informed consent for participation in the study.

The inclusion criteria were as follows: being a registered DGW under the Cooking Gas Workers’ Association Enugu or personnel on nominal roll of the Works Department of UNEC and UNTH; age range between 18 and 50 years; and absence of cardiopulmonary diseases, physical disability, psychiatric and psychological disorders. Potential participants who smoke and those with prior diagnosis of idiopathic-pain; complex regional pain syndrome; chronic stress and anxiety disorders; retinopathy; and neurological, vascular, cardiac, renal, respiratory, and rheumatoid diseases were excluded from the study.

A minimum sample size of 96 was calculated at 95% power, 0.05 level of error, an effect size of 0.73 with an equal allocation ratio (1:1), under two tails independent t-test using G* power 3.0.10 software. However, a total of 100 (50 in each group) participants were recruited for this study. Ethical approval for this study was obtained from the Research and Ethics Committee of the University of Nigeria Teaching Hospital, Enugu State, Nigeria.

### Research instruments

The research instruments used in this study include stadiometer and weighing scale for assessing the height and weights of the participants as well as the estimation of their body mass index (BMI). Others were sphygmomanometer and stethoscope (for assessing blood pressure), stopwatch, numerical pain rating scale (NPRS), the Nordic Musculoskeletal Questionnaire, and a self-developed but validated questionnaire for assessing work-related risk factors.

Nordic Musculoskeletal Questionnaire (NMQ) was developed by Kuorinka et al. [13] to assess regional and general MSD. It contained a body map to indicate nine symptom sites: neck, shoulders, upper back, elbows, low back, wrist or hands, hips or thighs, knees, and ankle feet. The questionnaire also captured data on the incidence of musculoskeletal symptoms in the last 7 days and 12 months. It also obtained information on whether the musculoskeletal symptoms had prevented the respondent from carrying out normal daily activities and whether the respondent had consulted a physician for MSD in the last 1 year. The test-retest reliability of NMQ is about 0.8. Its sensitivity ranges between 66 and 92%, and its specificity is between 71 and 88% [13, 14].

The present researchers developed a two-part, face- and content-validated, semi-structured questionnaire to collect data on the participants’ demographics and work-related WMSS risk factors. Part A was designed to record the participants’ age, sex, height, weight, BMI, and cardiopulmonary parameters. Part B consisted of eight items. Items 1 to 4 were open-ended questions designed to collect information on duration of service (years), work frequency (days per week), daily work duration (hours), and daily sleep duration (hours). Items 5 to 8 were dichotomous (yes or no) questions designed to determine whether the respondents exercised regularly, used personal protective equipment (PPE) while working, had training on ergonomics, and observed shift- or off-duty and break periods. The validation panel consisted of five experts in MSD; they interacted twice through the Delphi method of e-mail exchanges before arriving at the consensus on the face and content validity of the instrument.

### Procedures and measurements

Firstly, the protocol for the study was explained to the participants, and their informed consents were sought and obtained. The researcher-made questionnaire was administered to the participants, and the completed questionnaire was retrieved and stored.

Secondly, the participants’ height (m) and weight (kg) were measured using a standard BMI apparatus (RGZ-120, made in China; weight/[height]^2^ = BMI) and protocol [15]. Musculoskeletal pain intensity was obtained using NPRS, which rates pain from 1 (least pain) to 10 (worst pain). For participants with multiple painful regions, the intensity of the most painful region was recorded.

Thirdly, the cardiovascular parameters were measured three times, and the median score was recorded. The systolic and diastolic blood pressure (mmHg) were measured after 5 min of rest, using mercury sphygmomanometer (Accoson mercury, made in the United Kingdom) and stethoscope (Litman, made in the United States). The blood pressure was measured from the left brachial artery in a sited position with the left elbow flexed at the level of the heart. Then, the resting heart rate (RHR) in beats per minute (bpm) and respiratory rate (RR) in cycles per minute (cpm) were measured and recorded using a stopwatch (Kadio, made in China).

Finally, the NMQ was administered to assess the prevalence of WMSS. The questionnaire presented a figure of the human body with nine anatomical regions to assist the participants to mark the corresponding regions of their body on which they had MSD symptoms (aches, pains, discomfort or numbness) in the last 7 days and the last 12 months.

### Data analysis

The data obtained were processed and analysed using statistical package for social sciences (SPSS) version 20 software. Descriptive statistics – frequency (percentage), mean ± standard deviation and median (range) were used to summarise the demographic characteristics and prevalence of WMSS among the participants. Test of normality for the continuous variables measured on a ratio scale was done using Shapiro-Wilks test. Independent *t*-test and Mann-Whitney *U* test were used to respectively compare normally and non-normally distributed variables. The Chi-square test was used to compare the categorical variables and to test for association between WMSS and some selected risk factors within the two groups. Multilevel logistic regression modelling was used to predict the occurrence of WMSS. The level of significance was set at *p* ≤ 0.05.

## Results

### Demographic details of the domestic gas workers (DGWs) and staff of works department (SWD)

The study involved 100 participants who were divided into two study groups (DGWs and SWD), each with equal number (50) of participants. Table 1 presents the demographic characteristics of all participants. Each group comprised 8(16%) women and 42(84%) men, with no significant differences in sex distribution between the groups (χ^2^ < 0.001, *p* = 1.000). Similarly, there was no significant difference between the mean ages of the DGWs and SWD (*t* = − 0.360, *p* = 0.720).

**Table 1:** Demographic Characteristics of the Domestic Gas Workers and Staff of Works Department (*n* = 100)

Variable	*f* (%) / Mean ± SD / Median [Range]			M.D	χ^2^ /	*p*-value
	Total (*n* = 100)	DGW (*n* = 50)	SWD (*n* = 50)		t-score	
Gender						
Male	84 (84)	42 (84)	42 (84)	–	0.001^a^	1.000
Female	16 (16)	8 (16)	8 (16)	–		
Age (years)	31.95 ± 8.80	31.74 ± 8.24	32.16 ± 0.60	−0.42	−0.360^b^	0.720
Height (m)	1.70 [0.23]	1.70 [0.18]	1.70[0.23]	–	–	0.233
Weight (kg)	76.34 ± 8.29	74.36 ± 9.11	78.32 ± 6.91	−3.96	−2.448^b^	0.016*
BMI (kg/m^2^)	25.66 [18.01]	25.37 [18.01]	25.95 [14.68]	–	–	0.023*
SBP (mmHg)	120.00 [50.00]	110.00 [40.00]	120.00 [50.00]	–	–	0.002*
DBP (mmHg)	79.00 [30.00]	74.00 [35.00]	80.00 [30.00]	–	–	0.443
RR (cpm)	22.00 [12.00]	22.50 [7.00]	21.50 [12.00]	–	–	0.105
RHR (bpm)	74.00 [78.00]	74.00 [35.00]	73.00 [68.00]	–	–	0.675
Duration of service (years)	2.00 [39.92]	1.75 [39.92]	5 [39.50]	–	–	< 0.001*
Work duration (hours/day)	8.00 [8.00]	10.00 [7.00]	8.00 [2.00]	–	–	< 0.001*
Sleep duration (hours/day)	6.00 [2.00]	6.00 [1.00]	5.00 [2.00]	–	–	0.859

*f* frequency, % percentage, *DGW* Domestic Gas Workers, *SWD* Staff of Works Department, *M.D.* Mean Difference, *SD* Standard Deviation, *BMI* Body Mass Index, *SBP* Systolic Blood Pressure, *DBP* Diastolic Blood Pressure, *RR* Respiratory Rate, *RHR* Resting Heart Rate

^a^ = χ^2^-stastistics

^b^ = *t*-statistics

* = significant at *p* < 0.05

### Prevalence of WMSS among the domestic gas workers (DGW) and staff of works department (SWD)

The 12-month general WMSS prevalence for the entire population was 62% (DGWs = 86%, SWD = 38%). The general WMSS prevalence among DGWs was significantly higher than that in the SWD (*X*^*2*^ = 24.45, *p* < 0.001). The majority of the DGWs (50%) had WMSS in at least 3 regions of their body, whereas only 6% of SWD had WMSS in 3 or more regions of their body. The regional WMSS occurrence reported in Table 2 revealed that the low back (54%) and shoulder (52%) were the most affected sites among the DGWs, whereas the hips/thighs (20%) were the most affected body part among the SWD. When compared with the SWD, the DGWs had significantly higher prevalence of shoulder WMSS (*X*^*2*^ = 18.38, *p* < 0.001), upper back (*X*^*2*^ = 18.78, *p* < 0.001), elbow (*X*^*2*^ = 12.71, *p* < 0.001) and lower back (*X*^*2*^ = 33.53, *p* < 0.001). However, no significant difference was observed in the WMSs prevalence of the neck (*X*^*2*^ = 0.71, *p* = 0.68), wrist and hand (*X*^*2*^ = 2.17, *p* = 0.269), hip and thigh (*X*^*2*^ = 0.51, *p* = 0.64), knees (*X*^*2*^ = 0.001, *p* = 1.00), and ankle/foot (*X*^*2*^ = 0.344, *p* = 1.00).

**Table 2:** Comparison of the Prevalence of Work Related Musculoskeletal Disorders among Domestic Gas Workers and Staff of Works Department (*n* = 100)

Variable	*frequency* (*%*)			*χ*^*2*^-score	*p*-value
	DGW (*n* = 50)	SWD (*n* = 50)	Total (*n* = 100)		
**General WMSD Prevalence**	43 (86)	19 (38)	62 (62)	24.45	< 0.001*
**Number of WMSD regions**					
1	9 (18)	12 (24)	21 (21)	39.16	< 0.001*
2	10 (20)	5 (10)	15 (15)		
3	13 (26)	2 (4)	15 (15)		
> 4	12 (24)	1 (2)	13 (13)		
**Regional WMSD Prevalence**					
Neck WMSD	2 (4)	4 (8)	6 (6)	0.71	0.680
Shoulder WMSD	26 (52)	6 (12)	32 (32)	18.38	< 0.001*
Upper back WMSD	18 (36)	1 (2)	19 (19)	18.78	< 0.001*
Elbow WMSD	19 (38)	4 (8)	23 (23)	12.71	< 0.001*
Wrist/hand	6 (12)	2 (4)	8 (8)	2.71	0.269
Lower back	27 (54)	1 (2)	28 (28)	33.53	< 0.001*
Hips/thighs	13 (26)	10 (20)	23 (23)	0.51	0.640
Knees	4 (8)	4 (8)	8 (8)	0.001	1.000
Ankles/foot	1 (2)	2 (4)	3 (3)	0.34	1.000
**Activity limitation**	8 (16)	1 (2)	9 (9)	5.98	0.031*
**Has seen a physician in the last 1-year**	9 (18)	2 (4)	11(11)	5.01	0.051
**Pain intensity** (NPRS)				25.02	< 0.001*
None	9 (18)	32 (64)	41 (41)		
Low (1–4)	11 (22)	4 (8)	15 (15)		
Moderate (5–6)	25 (50)	8 (16)	33 (33)		
High (7–10)	5 (10)	6 (12)	11 (11)		

*DGW* Domestic Gas Workers, *SWD* Staff of Works Department, *WMSD* Work-Related Musculoskeletal Disorders, *NPRS* Numerical Pain Rating Scale

* = significant (2-tailed) test at *p* < 0.05

A good number of the participants described their pain as moderate intensity on the numerical pain rating scale (DGWs = 50%, SWD = 16%). Pain intensity was significantly higher in DGWs group than in the SWD group (*X*^*2*^ = 25.02, *p* < 0.001).

### Work-related risk factors of WMSS among domestic gas workers (DGW) and staff of works department (SWD)

A significantly greater number of DGWs (76.0%) reported being exposed to higher work frequency (≥ 6 days per week) compared to the few reports from the SWD (6%), (*X*^*2*^ = 82.02, *p* < 0.001). All the participants in the SWD were government-employed workers; most of whom (90%) worked only on weekdays (maximum of 5 days per week), while the rest had shift-duty*,* which sometimes falls into the weekend. Shift-duty was significantly higher among the SWD than the DGWs (*X*^*2*^ = 10.75, *p* = 0.002). Compared to the SWD, majority of the DGWs did not use PPE such as facemask (84% vs 30%) and gloves (84% vs 2%), had no training on lifting techniques (68% vs 10%), and did not exercise regularly (70% vs 42%). A significantly greater proportion of the SWD underwent lifting technique training (*X*^*2*^ = 96.24, *p* < 0.001), as well as used PPE of facemask (*X*^*2*^ = 100.0, *p* < 0.001) and gloves (*X*^*2*^ = 96.09, *p* < 0.001) than the DGWs as shown in Table 3.

**Table 3:** Comparison of Work-related risk factors of WMSD among Domestic Gas Workers and Staff of Works Department (*n* = 100)

Variables	*frequency* (*%*)			*χ*^*2*^-score	*p*-value
	DGW (*n* = 50)	SWD (*n* = 50)	Total (*n* = 100)		
**Work frequency** (days/week)					
≤ 4	0 (0)	0 (0)	0 (0)	82.02	< 0.001*
5	0 (0)	45 (90)	45 (45)		
6	38 (76)	3 (6)	41 (41)		
7	12 (24)	2 (4)	14 (14)		
**Rest break** (compliance)					
No	48 (96)	44 (88)	92 (92)	2.17	0.269
Yes	2 (4)	6 (12)	8 (8)		
**Training on lifting technique**					
No	34 (68)	5 (10)	39 (39)	96.24	< 0.001*
Yes	16 (32)	45 (90)	61(61)		
**Duty shifts**					
No	45 (90)	31(62)	76 (76)	10.75	0.002*
Yes	5 (10)	19 (38)	24 (24)		
**Use of PPE(face mask)**					
No	41(84)	15(30)	56(56)	100.00	< 0.001*
Yes	9(18)	35(70)	44(44)		
**Use of PPE(gloves)**					
No	41(84)	1(2)	42(42)	96.09	< 0.001*
Yes	9(18)	49(98)	58(58)		
**Exercise status**					
No	35 (70)	21 (42)	56 (56)	7.95	0.005*
Yes	15 (30)	29 (58)	44 (44)		

*DGW* Domestic Gas Workers, *SWD* Staff of Works Department, *WMSD* Work-Related Musculoskeletal Disorders, *PPE* Personal Protective Equipment (face mask, hand glove)

* = significant (2-tailed) test at *p* < 0.05

### Association between WMSS and selected variables among domestic gas workers (DGWs) and staff of works department (SWD)

There was a significant association between general WMSS and each of diastolic blood pressure level (χ2 = 20.79, *p* = 0.004), training on lifting techniques (χ2 = 2.36, *p* = 0.036), use of PPE (χ2 = 7.64, *p* = 0.022) and daily work duration (χ2 = 21.06, *p* = 0.007) among the DGWs. Among the SWD, gender (χ2 = 5.53, *p* = 0.027), work frequency (χ2 = 9.06, *p* = 0.011) and daily work duration (χ2 = 5.86, *p* = 0.05) were significantly associated with general WMSS as shown in Tables 4 and 5.

**Table 4:** Association between WMSD and selected demographic and cardiopulmonary variables among Domestic Gas Workers and Staff of Works Department (*n* = 100)

Variables	*χ*^*2*^*-statistics (p-value)*					
	General WMSD		Shoulder WMSD		Low Back WMSD	
	DGW (*n* = 50)	SWD (*n* = 50)	DGW (*n* = 50)	SWD (*n* = 50)	DGW (*n* = 50)	SWD (*n* = 50)
Age (years)	23.67 (0.250)	32.31 (0.149)	41.70 (0.003)*	23.17 (0.560)	36.00 (0.020)*	50.00 (0.002)*
Gender	0.04 (0.616)	5.53 (0.027)*	7.39 (0.008)*	1.52 (0.240)	8.00 (0.006)*	5.36 (0.160)
Height (m)	7.91 (0.849)	10.15 (0.602)	27.16 (0.012)*	10.97 (0.530)	25.00 (0.020)*	1.66 (1.000)
Weight (kg)	24.54 (0.268)	14.98 (0.777)	22.41 (0.376)	19.2 (0.500)	22.27 (0.380)	11.73 (0.930)
BMI	41.69 (0.571)	34.44 (0.678)	47.99 (0.314)	35.79 (0.617)	47.97 (0.310)	24.49 (0.960)
SBP (mmHg)	5.70 (0.457)	4.89 (0.769)	9.47 (0.149)	8.46 (0.389)	9.27 (0.159)	1.81 (0.986)
DBP (mmHg)	20.79 (0.004)*	7.27 (0.122)	12.60 (0.082)	0.93 (0.920)	15.10 (0.030)*	1.29 (0.862)
RR (cpm)	13.71 (0.057)	8.02 (0.331)	15.85 (0.027)*	2.91 (0.89)	14.40 (0.040)*	2.62 (0.917)
RHR (bpm)	3.41 (0.992)	10.26 (0.507)	14.22 (0.287)	11.16 (0.430)	14.04 (0.290)	2.90 (0.990)

*DGW* Domestic Gas Workers, *SWD* Staff of Works Department, *BMI* Body Mass Index, *SBP* Systolic Blood Pressure, *DBP* Diastolic Blood Pressure, *RR* Respiratory Rate, *RHR* Resting Heart Rate

* = significant (2-tailed) test at *p* < 0.05

**Table 5:** Association between WMSD and selected work-related risk factors among Domestic Gas Workers and Staff of Works Department (*n* = 100)

Variables	*χ*^*2*^*-stastistics (p-value)*					
	General WMSD		Shoulder WMSD		Low Back WMSD	
	DGW (*n* = 50)	SWD (*n* = 50)	DGW (*n* = 50)	SWD (*n* = 50)	DGW (*n* = 50)	SWD (*n* = 50)
Daily work duration	21.06 (0.007)*	5.86 (0.050)*	12.24 (0.141)	4.780 (0.090)	13.30 (0.101)	0.26 (0.880)
Break	0.34 (0.737)	0.06 (0.588)	2.26 (0.225)	0.93 (0.440)	2.44 (0.207)	0.14 (0.880)
Sleep duration	11.35 (0.125)	4.65 (0.795)	26.80 (< 0.001)*	4.19 (0.830)	31.3 (< 0.001)*	3.61 (0.890)
Work shift	0.90 (0.454)	0.22 (0.430)	0.14 (0.549)	2.37 (0.130)	0.08 (0.578)	1.66 (0.380)
Work frequency	2.57 (0.277)	9.06 (0.011)*	1.74 (0.499)	9.17 (0.100)	2.97 (0.226)	0.11 (0.940)
Year of service	34.77 (0.070)	20.84 (0.346)	22.10 (0.181)	22.01 (0.280)	22.68 (0.160)	50.0 (< 0.001)*
Lifting training	2.36 (0.036)*	1.67 (0.380)	0.00 (0.543)	7.48 (0.120)	0.05 (0.535)	50.00 (0.020)*
Use of PPE(gloves)	7.64 (0.022)*	1.67 (0.380)	3.80 (0.149)	0.13 (0.880)	3.48 (0.178)	0.02 (0.980)
Exercise status	3.49 (0.067)	0.88 (0.642)	5.51 (0.020)*	0.38 (0.670)	1.69 (1.610)	0.80 (0.670)

*DGW* Domestic Gas Workers, *SWD* Staff of Works Department, *PPE* personal protective equipment

* = significant (2-tailed) test at *p* < 0.05

Among the DGWs, there was a significant association between shoulder WMSS and each of age (χ2 = 41.70, *p* = 0.003), sex (χ2 = 7.39, *p* = 0.008), height (χ2 = 27.16, *p* = 0.012), respiratory rate (χ2 = 15.85, *p* = 0.027), sleep duration (χ2 = 26.80, *p* < 0.001), and exercise status (χ2 = 5.51, *p* = 0.020). Conversely, there was no significant association between these participants variables and shoulder WMSS among the SWD (*p* > 0.05) as shown in Tables 4 and 5.

Lastly, there was a significant association between low back WMSS and each of age (χ2 = 36.00, *p* = 0.020), sex (χ2 = 8.00, *p* = 0.006), height (χ2 = 25.00, *p* = 0.020), diastolic blood pressure (χ2 = 14.40, *p* = 0.040) and sleep duration (χ2 = 31.30, *p* < 0.001) among the DGW. Among the SWD, only age (χ2 = 50.00, *p* = 0.002) and years of service (χ2 = 50.00, *p* < 0.001) were significantly associated with WMSS around the low back region as shown in Tables 4 and 5.

### Predictors of WMSS among domestic gas workers (DGWs) and staff of works department (SWD)

A multi-level logistic regression was performed to ascertain the effects of sex, blood pressure, training on lifting technique, work frequency, and daily work duration on the likelihood that participants (DGWs and SWD) have musculoskeletal symptoms in any part of their body (general WMSS). The model for both groups was significant and explained 76.0 and 22% (Nagelkerke *R*^*2*^) of the variance in general WMSS and it correctly classified 92.0 and 70.0% of cases for the DGWs and SWD respectively. Higher diastolic blood pressure (OR = 1.29, *p* = 0.018) and working beyond 8 h per day (OR < 0.01, *p* = 0.028) were the independent predictors of general WMSS among the DGWs. Among the SWD, diastolic blood pressure (OR = 0.99, *p* = 0.012) and female gender (OR = 6.47, *p* = 0.032) were the independent predictors of general WMSS as shown in Table 6.

**Table 6:** Trimmed Regression Models for Predicting WMSD among Domestic Gas Workers and Staff of Works Department (*n* = 100)

Independent Variables	*Odds Ratio (p-value)*					
	General WMSD		Low back WMSD		Shoulder WMSD	
	DGW (*n* = 50)	SWD (*n* = 50)	DGW (*n* = 50)	SWD (*n* = 50)	DGW (*n* = 50)	SWD (*n* = 50)
Diastolic Blood Pressure (mmHg)	1.29 (0.018)*	0.99 (0.012)*	–	–	–	–
work duration (> 8 h/day)	< 0.001 (0.028)*	–	–	–	–	–
Gender (female)	1.13 (0.943)	6.47 (0.032)*	10.26 (0.021)*	0.001 (0.997)	6.98 (0.046)*	0.22 (0.118)
Age (years)	–	–	1.07 (0.062)	0.97 (0.816)	1.04 (0.276)	0.95 (0.299)
Sleep duration (< 6 h)	–	–	0.56 (0.004)*	0.93 (0.942)	0.73 (0.044)*	1.07 (0.791)
Exercise Status (poor)	–	–	–	–	0.15 (0.013)*	3.02 (0.331)
Nagelkerke R^2^	0.76	0.22	0.36	0.96	0.33	0.68
Classification	92.00%	70.00%	82.00%	98.00%	70.00%	88.00%

*WMSD* Work Related Musculoskeletal Disorder, *DGW* Domestic Gas Workers, *SWD* Staff of Works Department

* = significant (2-tailed) test at *p* < 0.05

Another significant model for predicting low back WMSS among these cohorts had age, sex, sleep duration and exercise status as its independent variables and explained 36.0 and 96.0% (Nagelkerke R^2^) of the variance in WMSS of the low back and also correctly classified 82.0 and 98.0% of these cases for the DGWs and SWD, respectively. Being a female (OR = 10.26, *p* = 0.021), and decreased sleep duration below 6 h per day (OR = 0.56, *p* = 0.004) were the significant independent predictors of low back WMSS among the DGWs. Contrarily, none of these variables independently predicted low back WMSS among the SWD (*p* > 0.05) as shown in Table 6.

Lastly, a model for predicting WMSS of the shoulder region included gender, age, sleep duration, and exercise status as the independent variable. This model explained 33.0 and 76.0% (Nagelkerke R^2^) of the variance in WMSS of the shoulder as well as classified 68.0 and 88.0% of shoulder WMSD cases, respectively, for the DGWs and SWD. Female gender (OR = 6.98, *p* = 0.046), sleep duration less than 6 h per day (OR = 0.73, *p* = 0.044), and poor exercise habit (OR = 0.15, *p* = 0.013) independently and significantly predicted shoulder WMSS among the DGW. These variables failed to significantly predict shoulder WMSS among the SWD (*p* > 0.05) as shown in Table 6.

## Discussion

The result of the present study showed a significantly higher WMSS prevalence among DGWs than SWD. The data showed that DGWs were twice more prone to developing WMSS than their counterparts in works departments of universities. Personal factors (such as sleep habit and experience) and occupational factors (such as work duration, work frequency, training and use of PPE) were found to differ between the groups, each time DGWs had a higher risk ratio of WMSS than the SWD. A similarly high WMSS prevalence was reported among gas cylinder handlers in Taiwan [6] and was found to be higher than that of the general Taiwanese working population [16]. Also, a significantly greater proportion of the DGWs experienced higher pain intensities (moderate and severe) than the SWD. The present authors opined that the continual and cumulative exposure of the DGWs to WMSS risk factors such as prolonged work duration, poor sleep habits, and increased non-use of PPE may have worsened their WMSS. AlNekhilan et al. [17] supported the thesis that continual exposure of workers to job strain without rest period, worsens their pre-existing WMSS. Therefore, facility managers and administrative officers are advised to allow shift duty, casual leaves, health leaves, and other breaks that could help their staff to recover from job stress or seek health care, when required.

Our findings showed that the average work duration and frequency of DGWs (10 h daily/6 days per week, i.e. 60 h) was almost twice the period (7 h daily/5 days per week, i.e. 35 h) reported by the SWD. Most of the DGWs in Enugu operate between 7:00 am and 8:00 pm, similar to petrol station attendants in Nigeria [18]. Comparatively, the majority of the SWD work either on a day shift (8:00 am to 4:00 pm) or night shift (4:00 pm to 8:00 am). Working beyond 8 h was a significant predictor of WMSS of the low back and shoulders among the DGWs. Work settings with longer daily work duration are consistently associated with poor health outcomes. Soe et al. [19] reported that working hours significantly associated with WMSS on Myanmar migrant workers. This report also concurred with another study that found a significant association between long working hours and prevalence of WMSS among Korean workers [10]. Prolonged job duration constitutes a risk of musculoskeletal disorders through several mechanisms. First, it enhances the release of stress hormones such as cortisol, cathecholamines, and vasopressin. Those hormones are capable of inducing muscle fatigue and predisposing the myotomes to microtrauma [20]. Secondly, prolonged job duration increases exposure time to physical demands such as awkward postures, repetitive movements, and heavy lifting that may lead to musculoskeletal injury [4]. Finally, it can cause a relative decrease in rest, leisure, and recovery time. It is therefore recommended that the work set up in domestic gas stations in Nigeria should be restructured to enable shifts of less than 8 h per day and a rest break during shift duty.

Furthermore, there was an inverse relationship between daily work duration and sleep duration. It seems that workers who put in more hours at work have less sleep time. Sleep duration was found to be a significant predictor of WMSS among the DGWs. Sleep deprivation sets the stage for tissue injury due to increased inflammatory markers such as cortisol (stress hormone), and prostaglandins [21]. After a day’s work, there is an increase in stress hormones such as cortisol [22], good sleep helps to modulate these hormones to optimal body functioning level [23]. Therefore, sleep deprivation, leads to an imbalance in these hormone levels and the body tends to malfunction. Moreover, inadequate sleep inhibits the release of human growth hormone [24] that is responsible for repairing damaged tissues thereby prolonging tissue healing time. It is therefore recommended that manual material handlers similar to those of DGWs should sleep for at least 6 h per day to encourage tissue healing of the microtrauma from the day’s work and prevent inflammatory tissue injury.

The need for continuous ergonomics training on lifting techniques for DGWs cannot be overemphasised. A piece of historical evidence has shown that carrying out a task without training imposes musculoskeletal stress with a detrimental effect on human anatomy and physiology [25]. Additionally, PPE such as gloves and boots can help in preventing direct contact forces capable of causing soft tissue injuries. Similar to the present study, scholars have proposed that compliance with the use of PPE reduces the risk of WMSS [26]. Moreover, the use of assistive devices such cylinder trolley and forklift can reduce the physical demands on DGWs.

In terms of body regions, WMSS was most common in the low back, and shoulders among the DGWs but highest around the hips and thighs among the SWD. This outcome appears congruent with the job description of these two groups. Task analysis for DGWs revealed stationary lifting of heavy cylinders and short distance transfer. These activities engage the antigravity muscles of the shoulders and the lower back stabilisers and continuous exposure to these factors may culminate in a shoulder and low back WMSS. On the other hand, the SWD appear to engage more in routine transfer of lighter objects to distant substations, with resultant overuse of the ambulatory (hip and thigh) muscles. A review of WMSS by the National Institute of Occupational Safety and Health (in the USA) found strong evidence that low back disorders are related to forceful lifting and weight of the load lifted [27] as was commonly seen among the DGWs. Based on these findings, lumbar core strength and stability exercises are recommended for DGWs while passive stretching and strengthening of the hip and thigh muscles are recommended for the SWD.

Furthermore, the present study identified a few demographic and work-related risk factors associated with the occurrence of general WMSS among the participants. There was a preponderance of male in both groups, presumably, due to the manual handling demands of these jobs. Similarly, higher proportion of male workers have been reported in other jobs requiring manual material handling such as construction work [28, 29], commercial driving [30, 31], and farming [32]. Among the SWD, significant associations were observed between general WMSS and each of gender, work frequency, and diastolic blood pressure. The Works Department in most tertiary institutions in Nigeria have various subunits such as carpentry, welding, wiring, construction, automobile, plumbing, environmental services, and general maintenance [11]. These manual and high energy demanding tasks may be unsuitable for the female workforce, considering some socio-cultural and physiological barriers. Therefore engaging female workers in these tasks may heighten the risk of WMSD among this cohort. In addition, being a female was reported as a predictor of general WMSS among SWD in this current study. This appears to be corroborated by the reports of Park et al. [33] who in their study among Korean workers reported that sex difference in WMSD depends on the different work sectors. Aside from the physical job demands, Josephson et al. [34] went further and opined that being a woman can be a risk factor for WMSD independent of the exposure situation. In a study carried out among workers in New Zealand, females had a significantly higher WMSD prevalence compared to males [35]. From the authors’ anecdotal knowledge, home chores and childcare imposes additional musculoskeletal stress on the typical Nigerian woman beyond the work environment. It is therefore recommended that extra attention should be paid to the female workers. Labour unions and government agencies should ensure that antenatal and maternity leaves are granted as prescribed in labour regulations. Duty rosters can be adjusted to accommodate workers who have other family and social roles such as childcare, school run, and home upkeep. Moreover, these work settings should enrol their staff in a health insurance scheme and ensure regular medical checks among the personnel.

The report of general WMSS among the DGW was associated with higher diastolic blood pressure, inadequate training on proper lifting techniques, and noncompliance with the use of PPE. Conversely, the SWD were not affected by these factors. This could be because the SWD worked in a formal setting (university and hospital environments), with access to healthcare, continuous professional training, and under firm occupational guidelines and supervision. Johansson et al. [36] reported that increased job stress among Swedish drivers correlated with elevated diastolic blood pressure and a higher prevalence of musculoskeletal pain. More so, diastolic blood pressure was a predictor of WMSS among the study participants. It is, therefore, necessary to regularly evaluate the blood pressure of workers as a direct measure of job strain and by extension, a correlate of musculoskeletal symptoms especially among workers with similar job tasks as DGWs.

There was no significant association between general WMSS and exercise status; however, poor exercise habit was an independent predictor of shoulder WMSS among the DGWs. The job task of the DGWs was seen to be characterized by heavy lifting and carrying of filled gas cylinders. These activities expose the shoulder joints to repeated traction forces that may increase shoulder instability and thus result in shoulder MSDs. To counteract this effect, it is required that the shoulder muscles especially the deltoid and rotator cuff muscles (supraspinatus, infraspinatus, teres minor, and subscapularis) should be regularly strengthened through resistant (muscle building) exercises. The DGWs who were unaware of this job risk factor, shoulder biomechanics, and benefits of shoulder muscle strengthening exercises, may present with shoulder WMSS. Serra et al. [37] indicated that physical exercise helped to reduce the risk of WMSS in a (Brazilian) workplace when compared with workers who did not engage in physical exercise and opined that exercise prepares workers against strenuous musculoskeletal demands. Workers should have a recreational period when they can engage in muscles-specific structured (resistant) exercises.

### Limitations

The participants were not randomly selected which could lead to distribution bias – affecting the generalisability of the findings. We were not able to control for possible extraneous variables such as domestic and recreational risk factors of WMSS.

## Conclusion

There is a high prevalence of WMSS among workers in domestic gas stations, significantly higher than those of their counterparts in the works departments of tertiary institutions. Low back and shoulder WMSS are more common among DGWs while thigh and hip WMSS are the most frequent WMSS among SWD. Stress indicated by high diastolic blood pressure, working greater than 8 h per day, female gender, sleep deprivation beyond 6 h per day, and poor exercise habits are the independent risk factors of WMSS among these cohorts. Therefore, SWD and DGWs require break and leave periods, PPE, assistive devices, exercise, medical check-up, and ergonomically designed workplace. A comprehensive and continuous ergonomics evaluation and training for both managerial and non-managerial staff of domestic gas stations, and institutional works departments is recommended. Also, the labour unions in Nigeria should ensure that workers in these sectors operate within the confines of healthy occupational guidelines.

## Data Availability

The questionnaire used and datasets analysed during the current study are available from the corresponding author on reasonable request.

## Abbreviations

BMI: Body Mass Index
DBP: Diastolic Blood Pressure
DGW: Domestic Gas Workers
MSD: Musculoskeletal Disorders
NMQ: Nordic Musculoskeletal Questionnaire
NPRS: Numerical Pain Rating Scale
PPE: Personal Protective Equipment
RHR: Resting Heart Rate
RR: Respiratory Rate
SBP: Systolic Blood Pressure
SPSS: Statistical Package for Social Sciences
UNEC: University of Nigeria Enugu Campus
UNTH: University of Nigeria Teaching Hospital
USA: United States of America
WMSS: Work-related Musculoskeletal Symptoms
